# Influence of Radio Frequency Heating on the Pasteurization and Drying of Solid-State Fermented *Wolfiporia cocos* Products

**DOI:** 10.3390/foods11121766

**Published:** 2022-06-15

**Authors:** Yu-Fen Yen, Su-Der Chen

**Affiliations:** Department of Food Science, National Ilan University, Number 1, Section 1, Shen-Lung Road, Yilan City 26041, Taiwan; abcz550068@gmail.com

**Keywords:** radio frequency (RF), pasteurization, drying, *Wolfiporia cocos*, solid-state fermentation

## Abstract

Rice bran and soybean residue are high in nutrients and active ingredients. They are used as media in the solid-state fermentation of *Wolfiporia cocos*. They not only reduce raw material costs, but also raise the economic value and applications of soybean residues and rice bran. After 30 days of fermentation, the moisture content (w.b.) of the *W. cocos* product was approximately 40%, requiring it to be pasteurized and dried later. The objective of this research is to use radio frequency (RF) rapid heating technology to pasteurize and dry the solid-state fermented product. A 500 g bag of solid-state fermented *W. cocos* product took only 30 and 200 s at the RF electrode gap of 15 cm to pasteurize and reduce the moisture content (w.b.) below 15%, respectively; therefore, the methods can be used instead of the traditional 60 min autoclave sterilization and 100 min hot air drying at 45 °C. After RF treatment, the fermented *W. cocos* product was white, indicating that browning was prevented; the product contained 5.03% mycelium, 9.83% crude polysaccharide, 4.43% crude triterpene, 3.54 mg gallic acid equivalent/g dry weight (DW) of total polyphenols, and 0.38 mg quercetin equivalent/g DW of flavonoid contents and showed a good antioxidant capacity.

## 1. Introduction

*Wolfiporia cocos* is a medicinal and edible fungi that is mainly harvested in China. Polysaccharides, triterpenoids, and bioactive compounds are abundant in *W. cocos*. The current demand for fungal health foods is high, with their market growing each year. If grains can be used as solid-state fermented media to provide suitable carbon and nitrogen sources for *W. cocos*, the mycelial growth and production of biologically active metabolites can be promoted [1]. The biofunctions of *W. cocos* include tumor inhibition [2], immunity improvement [3], and anti-inflammatory, antiaging, hypoglycemic [4], hypolipidemic, antibacterial [5], and antioxidant capacities. Soybean residues from soybean milk production can be rapidly dried by radio frequency (RF) energy [6], and rice bran from milled brown rice can be stabilized by RF heating [7]. When soybean residues and rice bran are mixed in a 1:1 ratio to form a solid-state medium for *W. cocos* fermentation, the cost of the process is reduced and a mycelial product rich in polysaccharides and triterpenoids is produced.

RF treatment is a type of dielectric heating. When food is placed between parallel top and bottom electrodes, the polar water molecules and charged ions in food can absorb electromagnetic radiation to generate heat via dipolar polarization and ionic movement. As a result, RF heating can overcome the issues caused by heat conduction and the convection of hot air heated from the outside to the inside, and RF energy provides volumetric and more uniform overall heating, deep penetration, and a moisture self-balance effect [8,9,10]. Because RF heating is an emerging processing technology, RF equipment has been used to study the rapid sterilization and drying of agricultural products or food [11].

The dielectric properties of vegetable powders, such as onion, chill, broccoli, tapioca flour, and potato starch, decrease with frequency and compacted density and increase with moisture content or temperature. The RF heating rates of vegetable powders, which range from 0.56 to 2.12 °C/s, are linearly related to moisture and the dielectric loss factor. RF technology has a fast-heating rate and a deep penetration depth, indicating that it may be an effective method for quickly pasteurizing dried vegetable powder, while maintaining a high product quality [12]. By intermittently rearranging layers during hot-air-assisted RF drying, carrot quality and heat uniformity are improved.

RF heating has been applied in food pasteurization. After 90 s of RF treatment, the reductions in *Salmonella* Typhimurium and *Escherichia coli* O157:H7 in creamy or chunky peanut butter were greater than 4 log CFU/g, and food quality was not affected [13]. The pathogens in black and red peppers were significantly reduced by RF heating for 50 and 40 s, respectively [14]. Heating prepackaged white bread to 58 °C or higher using combined RF and hot air treatment resulted in 4 log reductions in *Penicillium citrinum* spores and extended its storage time [15]. RF assisted the thermal processing pasteurization of low moisture content food, such as egg-white powder [16], powder infant formula milk [17], and walnut shells [18]. RF heating selectively killed the pathogens without damaging the food product due to the larger difference in the dielectric loss factor between target microorganisms and host foods [10,19].

Zhou and Wang [9] thoroughly overviewed recent advances in the RF drying of food and agricultural products. Fresh macadamia nuts [20] and walnuts [21] were dried using hot-air-assisted RF drying to significantly reduce drying time compared with drying only with hot air. Furthermore, the layer arrangement of carrot slices improved heat uniformity and quality produced by hot-air-assisted RF drying [22]. In addition, the RF vacuum system was controlled at 0.02 MPa, the final temperature of kiwi fruits was 60 °C, the drying time was reduced by 65% compared with hot air drying at 60 °C, and the quality of the RF vacuum-dried kiwi fruits was higher [23]. The total drying time of chicken powders was reduced, and the umami flavor of chicken powder was improved by vacuum RF drying [24].

After 30 days of solid-state fermentation, *W. cocos* has to be pasteurized to stop the fermentation reaction, and then it can be dried for storage. Traditional sterilization is achieved by autoclaving and then drying by hot or cold air [25], which are time-and energy-intensive. Furthermore, long high-temperature treatments may destroy the active ingredients in solid-state fermented *W. cocos* products. Therefore, the objectives of this study are to investigate the suitability of RF heating for the pasteurization and drying of solid-state fermented *W. cocos* products. The quality attributes of the RF-treated products were analyzed with bioactive components and according to the color of the products, and then the results were compared with those produced by traditional autoclaving and hot air drying.

## 2. Materials and Methods

### 2.1. Materials

Soybean residues were obtained from Kuang Chuan Dairy Co., Ltd. (Taoyuan, Taiwan). Rice bran was purchased from Jiyuan Farm (Yilan, Taiwan). *Wolfiporia cocos* (BCRC 36022) was purchased from Bioresource Collection and Research Center (Hsinchu, Taiwan). Potato dextrose agar (PDA) and potato liquid broth (PDB) were purchased from Difco Co., Ltd. (Sparks, MD, USA). Gallic acid, quercetin, ascorbic acid, butylated hydroxyanisole (BHA), ethylenediaminetetraacetic acid (EDTA), 1,1-diphenyl-2-picryl hydrazyl (DPPH), ferrozine, ferrous chloride (FeCl_2_·4H_2_O), trichloroacetic acid (TCA), Folin–Ciocalteu phenol reagent, ergosterol standard, vanillin (C_8_H_8_O_3_), and perchloric acid were purchased from Sigma Chemical Company (St. Louis, MO, USA). We obtained 99% methanol, 95% ethanol, sodium carbonate (Na_2_CO_3_), potassium dihydrogen phosphate (KH_2_PO_4_), ferric chloride (FeCl_3_), glucose standard (C_6_H_12_O_6_), sodium hydroxide (NaOH), and a phenol solution from WAKO Pure Chemical Industries, Ltd. (Osaka, Japan). Acetate and sulfuric acids were purchased from Union Chemical Works (Taipei, Taiwan).

### 2.2. Equipment

A radio frequency with hot air equipment (40.68 MHz, 5 kW, Yh-Da Biotech Co., Ltd., Yilan, Taiwan), electric oven (Channel DCM-45, Yilan, Taiwan), high-speed grinder (RT-40, Sci-Mistry Co., Ltd., Yilan, Taiwan), horizontal laminar flow hood (4HT-24, Sage Vision Co., Ltd., New Taipei, Taiwan), constant temperature incubator (LM-600R, Yihder Co., Ltd., New Taipei, Taiwan), high-temperature steam vertical autoclave (Tommy SS-325, Tokyo, Japan), vacuum concentrator (Eyela Oil Bath Osb-2000, Tokyo, Japan), centrifuge (Hsiangtai Centrifuge, Yihua Company, New Taipei, Taiwan), high-speed batch top centrifuge (Hermle Z300, Wehingen, Germany), vortex mixer (Vortex Genie 2, Scientific Industries, Inc., New York, NY, USA), spectrophotometer (Model U-200l, Hitachi Co., Tokyo, Japan), electronic precision scale, infrared thermometer (TM-300, Tenmars Electronics Co., Ltd., Taipei, Taiwan), colorimeter (Hunter Lab, Color Flex, Hunter Associates Laboratory Inc., Reston, VA, USA), ultrasonic cleaner (DC-600H, Delta, Yuantuo Technology Ltd., Taichung, Taiwan), fiber optic thermometer (FOB100, Omega Engineering, Norwalk, CT, USA), thermal imaging camera (TIM03, Zytemp, Hsinchu, Taiwan), multifunctional infrared thermometer (Testo104-IR, Hot Instruments Co., Ltd., New Taipei, Taiwan), microwave extraction system (Bio-Promotion Co., Ltd., Taoyuan, Taiwan), and HPLC equipment (Waters Co., Milford, MA, USA), which included a Waters^TM^510 pump, Waters^TM^717 autosampler, Athena C18 column (4.6 × 250 mm, 5 μm), and Waters^TM^486 UV–Vis detector, were used in this study.

### 2.3. Sample Preparation

#### 2.3.1. Maintenance and Pre-Activation of *W. cocos*

*Wolfiporia cocos* (BCRC 36022) was inoculated on a PDA plate medium and cultivated in a 25 °C incubator for 7 days. The strain was cut into 1 cm^2^ pieces and the pieces were inoculated in a 500 mL flask with 150 mL of presterilized PDA medium at 25 °C and 150 rpm shaking for 7 d of pre-activation.

#### 2.3.2. Solid-State Fermentation of *W. cocos*

The RF-dried soybean residue [6] and RF-stabilized rice bran [7] were mixed as a 1:1 ratio to form a solid-state medium (40% moisture content) in a plastic bag, and then sterilized in a 121 °C autoclave. After cooling, 10 mL of the pre-activated *W. cocos* solution was inoculated into 500 g of the solid-state medium and cultured for 30 d at 25 °C in an incubator.

### 2.4. Pasteurizing and Drying Solid-State Fermented W. cocos Products

#### 2.4.1. RF Output Power Measurement

The polypropylene (PP) plastic bag (8 × 9 × 29 cm) with 500 g solid-state fermented *W. cocos* product was horizontally placed between two parallel electrode plates in the hot-air-assisted RF equipment, and the RF output power was measured for different electrode plate gaps. Because the maximum current and output power of the 5 kW, 40.68 MHz RF equipment were 1.6 A and 5 kW, respectively, the output current (I, A) was measured three times and the output RF power was calculated using the following formula:

power output (kW) = (I/1.6) × 5(1)

#### 2.4.2. RF Pasteurization

The solid-state fermented *W. cocos* products were heated by RF energy and samples were taken out every 10 s, and the surface temperature at three locations was measured with an infrared thermometer. The fermented products of various RF pasteurization times were inoculated on a PDA plate and cultured for 7 days in a 25 °C incubator to observe the lethal situation of *W. cocos*.

#### 2.4.3. Heating and Drying Curves of Solid-State Fermented *W. cocos* Products during Hot-Air-Assisted RF Drying

During RF drying, the polypropylene (pp) plastic bag of the solid-state fermented *W. cocos* product was left open to allow water vapor to evaporate. The sample was removed at periodic intervals (20 s) to measure the weight change with an electronic balance and surface temperature with an infrared thermometer to determine the drying curve and temperature profile during RF drying. Moreover, the center and internal (2 cm from the edge) temperatures of the sample were determined by inserting a fiber optic thermometer.

#### 2.4.4. Autoclaving and Hot-Air-Drying of the Solid-State Fermented *W. Cocos* Products

The PP plastic bag containing 500 g of solid-state fermented *W. cocos* product was heated in a 121 °C autoclave for 60 min, and then it was dried in a 45 °C hot-air-drying apparatus for 180 min. To determine the drying curve and temperature profile during hot air drying, the weight and temperature changes of the sample were measured at fixed time intervals.

### 2.5. Extraction of Solid-State Fermented W. cocos Products

The solid-state fermented *W. cocos* product was weighed to obtain a 2.5 g sample, and 50 mL of water or ethanol was added for 5 min microwave extraction, as described by Chen and Chen [25]. The hot water or ethanol extract was freeze-dried and dissolved to prepare the 20 mg/mL hot water or ethanol extract. 

### 2.6. Analytical Methods

#### 2.6.1. Moisture Content

Weigh 5 g of ground almonds in an aluminum dish and dry them in an oven at 105 °C for 12 h; then, remove and weigh them after reaching a constant.

Moisture content (wet basis) = (W_i_ − W_f_)/W_i_ × 100%. (2)

Solid content (wet basis) = W_f_/W_i_ × 100%. (3)

where W_f_ is the weight (g) of the dried sample and W_i_ is the initial weight (g) of the sample. 

The weight (W_t_) of sample during drying was measured, and then the dry basis moisture content of the sample during drying was calculated with the following equation:

Moisture content (dry basis) = (W_t_ − W_o_)/W_o_ (g water/g dry material) (4)

where W_t_ is the weight (g) of the sample at drying time t and W_o_ is calculated by W_i_ × solid content.

#### 2.6.2. Color Measurement

The color of sample was measured according to Chen et al. [7] with a color difference meter and standardized against a calibration white plate (X = 82.48, Y = 84.23, Z = 99.61; L* = 92.93, a* = − 1.26, b* = 1.17). The parameters determined were the degrees of lightness (L*), redness (+a*) or greenness (−a*), and yellowness (+b*) or blueness (−b*). All experiments were performed in six repetitions.

#### 2.6.3. Mycelium Determination of Solid-State Fermented *W. Cocos* Products

The ergosterol content represents mycelium in the mycelial fermentation product, following the process described by Chang et al. [26], with some modifications. The methanol supernatant from extract was filtered with a 0.22 µm membrane after centrifugation; its ergosterol content was determined by HPLC using a C18 column (250 × 4.6 mm) using 100% methanol as the mobile phase, a flow rate of 1 mL/min, an injection volume of 20 µL, and a UV detector at 282 nm.

#### 2.6.4. Crude Polysaccharide Analysis

With some modifications, the crude polysaccharide content was determined using the method described by Dubois et al. [27]. The hot water extract of the dried sample was mixed with four volumes of 95% ethanol and stirred the mixture vigorously, which was then collected by centrifugation at 5000× g for 20 min. The crude polysaccharide precipitate was washed twice with 95% (*v*/*v*) ethanol and then dried at 80 °C to remove residual ethanol. Then, the crude polysaccharides were dissolved in 1 mL of 1 N NaOH and determined the reducing sugar content of the supernatant using the phenol–sulfuric acid method. 

#### 2.6.5. Crude Triterpenoid Analysis

The crude triterpenoid content was measured according to Sun et al. [28] with some modifications. In an 80 °C dry bath incubator, the ethanol extract (0.1 mL) was evaporated to dryness. The dried extract was redissolved in 0.4 mL of 5% vanillin–acetic acid solution and 1 mL of perchloric acid solution at 60 °C. After a 15 min reaction, the extract was cooled to room temperature in an ice bath, and 5 mL of acetic acid was added. After 15 min of reaction, the absorbance at 548 nm was measured using a spectrophotometer to determine the triterpenoid content.

#### 2.6.6. Total Phenol Analysis

The concentration of total phenolic compounds was determined using the method described by Antolovich et al. [29], with some modifications. A 0.2 mL sample of ethanol extract was mixed with 1 mL of Folin–Ciocalteu phenol reagent and 0.8 mL of 7.5% Na_2_CO_3_. Following the addition, the solution was incubated in the dark for 30 min at room temperature. The absorbance of the solution was measured at 765 nm and compared to a gallic acid calibration curve (0–500 ppm) using a linear regression equation of y = 0.0046x + 0.22 (R^2^ = 0.993).

#### 2.6.7. Total Flavonoid Analysis

Total flavonoids were analyzed using a modified version of the method described by Christel et al. [30]. A 1 mL extract sample was mixed with 1 mL of 2% methanolic AlCl_3_. The solution was incubated at room temperature for 10 min. The absorbance of the solution was measured at 430 nm and the result was compared to a quercetin calibration curve (0–100 ppm) using a linear regression equation of y = 0.0206x + 0.0241 (R^2^ = 0.999).

#### 2.6.8. DPPH Radical Scavenging Activity

The DPPH free-radical-scavenging capacity of the extracts of the mycelial products obtained by solid-state fermentation was determined by following the method of Xu and Chang [31], with minor modifications. Briefly, 2 mL of the ethanol extract sample was mixed with a 2 mL ethanol solution of DPPH radical (final concentration was 0.2 mM). The mixture was vortexed vigorously for 1 min and then left at room temperature in the dark for 30 min. The absorbance of sample was then measured using a spectrophotometer at 517 nm against an ethanol blank. Ascorbic acid and BHA were used as controls. DPPH scavenging activity (%) = [1 − (ABS _sample_/ABS _control_)] × 100%.

#### 2.6.9. Ferric-Reducing Antioxidant Power (FRAP) Assay

The FRAP assay was carried out by following the method of Xu and Chang [31]. A 2 mL ethanol extract sample was added to a 1 mM FeCl_2_·4H_2_O (0.1 mL). The reaction was started by adding 0.25 mM ferrozine (0.2 mL). The mixture was vigorously shaken and allowed to stand for 10 min at room temperature. The absorbance was taken at 562 nm using a visible spectrophotometer. Ascorbic acid and EDTA were used as controls. Ferric reducing antioxidant power (%) = [1 − (ABS _sample_/ABS _control_)] × 100%.

#### 2.6.10. Reducing Power

A 2.5 mL ethanol extract sample was mixed with 0.2 mL of 0.2 M phosphate buffer and 2.5 mL of 1% potassium ferricyanide. The mixture was incubated at 50 °C for 20 min. Approximately 2.5 mL of 10% trichloroacetic acid was added to the mixture. The mixture was then centrifuged for 10 min at 3000 rpm and the supernatant (5 mL) was mixed with 5 mL of distilled water and 1 mL of 0.1% ferric chloride. The absorbance was monitored at 700 nm using a spectrophotometer. Ascorbic acid and BHA were used as controls [32].

### 2.7. Statistical Analysis 

The experimental results were presented as mean ± standard deviation (SD). A one-way analysis of variance (ANOVA) was performed and subsequently subjected to Duncan’s multiple range tests of treatment mean by using Statistical Analysis System (SAS 9.4, SAS Institute, Cary, NC, USA), and the significant level was set at 0.05.

## 3. Results and Discussion

### 3.1. RF Pasteurization Conditions of Solid-State Fermented W. cocos Products

**Figure 1:**
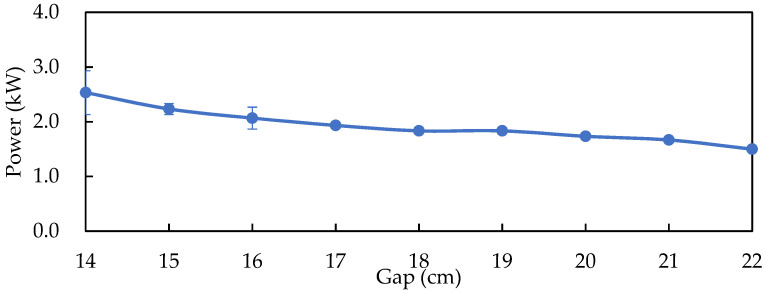
RF power output for different electrode gaps of 500 g solid-state fermented *W. cocos* product. Data are expressed as the mean ± SD (*n* = 3).

Figure 1 shows the RF output power of 500 g solid-state fermented *W. cocos* product placed between of two parallel electrode plates with different electrode gaps. The smaller the gap, the larger the RF output power; therefore, a gap for the electrode plate of 14, 15, and 16 cm was chosen for comparison of the temperature profile.

**Figure 2:**
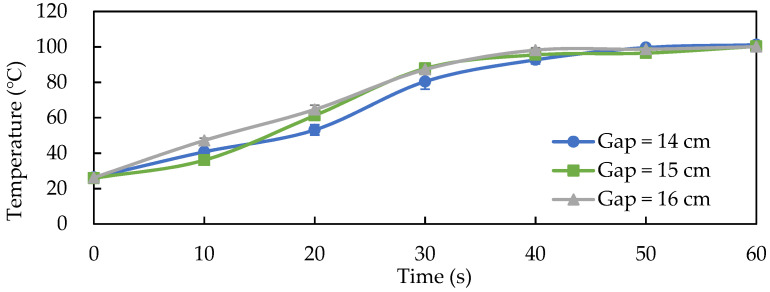
The temperature profile of 500 g solid-state fermented *W. cocos* mycelia product for different electrode gaps during RF pasteurization. Data are expressed as the mean ± SD (*n* = 3).

The average temperature profile of RF pasteurization (Figure 2) shows that the fermented product reached temperatures above 80 °C in less than 30 s with electrode plate gaps of 14, 15, and 16 cm. The temperature began to stabilize above 95 °C after 40 s of RF pasteurization. Because of the current output stability, we chose an electrode plate gap of 15 cm as the RF pasteurization condition for the solid-state fermented *W.* *cocos* products.

**Figure 3:**
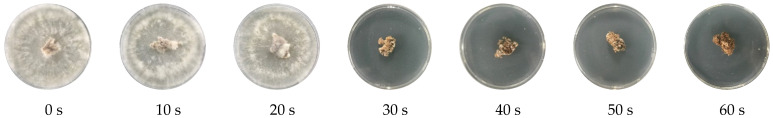
Center and internal temperature profiles of 500 g of solid-state fermented *W. cocos* product during RF with a gap of 15 cm, pasteurization of 1 min, and cooling.

The central and internal temperatures of the samples were substantially higher than the surface temperature. After 1 min of RF pasteurization and 10 min of room-temperature cooling, the central and internal temperatures of the fermented product remained above 80 °C (Figure 3), which exceeded the conditions for inactivating *W. cocos* (*W. cocos* died above 80 °C in previous experiments; data not shown in this paper).

**Figure 4:**
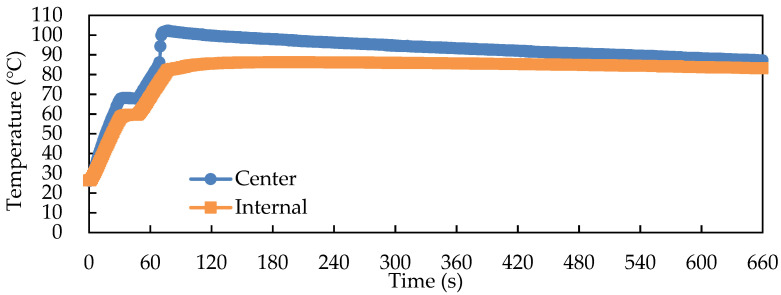
RF pasteurization with an electrode gap of 15 cm for solid-state fermented *W. cocos* products after 7 d of cultivation.

As a test of the pasteurization effect, solid-state fermented *W. cocos* products were RF pasteurized for 0 to 60 s and then incubated in PDA for 7 d (Figure 4). We observed that the fermented products only required 30 s of RF heating to achieve the pasteurization requirements. Because RF generates heat by making the polar water molecules in the sample rapidly rotate and oscillate, the heating rate of the samples during RF heating depended on the moisture content, loading, dielectric constant, and RF electrode gap.

In previous studies, white bread (37.1% moisture content) was treated for less than 5 min with HARF to reach 58 ℃ or higher, which reduced the fungi spores and thereby extended the storage time [15]. Walnut shells (9% moisture content) were heated to over 60 °C by RF pasteurization for 10 min to kill *Staphylococcus aureus.* RF heating inhibited nucleic acid metabolism, translation, cell membrane transport, and cell wall biosynthesis, which eventually led to the cell death of *Staphylococcus aureus* [18]. Therefore, RF pasteurization is suitable for low-moisture-content foods due to the large difference in the dielectric loss between the target microorganisms and host foods. The method selectively heated and killed the microorganisms without damaging the food product [19].

**Figure 5:**
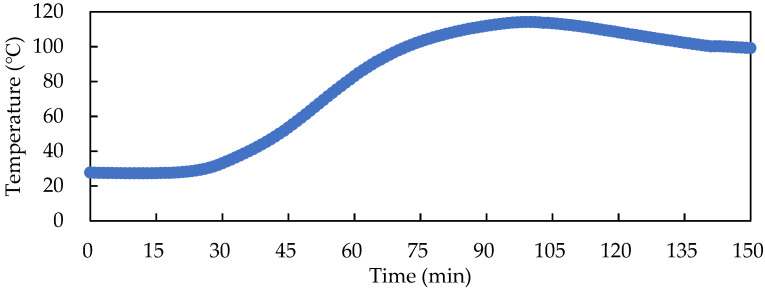
Center temperature profile of 500 g of solid-state fermented *W. cocos* product during 121 °C autoclaving for 60 min and cooling.

Figure 5 presents the temperature profile of the solid-state fermented *W. cocos* products produced by an autoclave at 121 °C for 60 min and a cooling process. The figure shows that the temperature rose very slowly from 0 to 30 min. At 100 min, the highest central temperature of 114.1 °C was reached, which then gradually decreased. This indicated that the heat transfer of the solid-state fermented *W. cocos* products was poor when using an autoclave.

**Figure 6:**
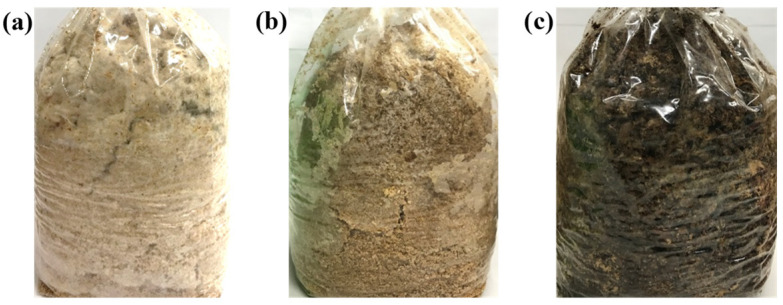
Effect of pasteurization method on the appearance of the solid-state fermented *W. cocos* products: (**a**) control, (**b**) RF heating for 30 s, and (**c**) 121 °C autoclaving for 60 min.

After 60 min of pasteurization in the traditional 121 °C autoclave, the solid-state fermented *W. cocos* products blackened due to the Maillard reaction (Figure 6). We observed no notable color change between the control group and the RF pasteurized solid-state fermented *W. cocos* product. This finding indicated that RF pasteurization can replace traditional autoclave treatment, reduce operation time, and improve product color preservation.

### 3.2. RF Drying Conditions of Solid-State Fermented W. cocos Products

**Figure 7:**
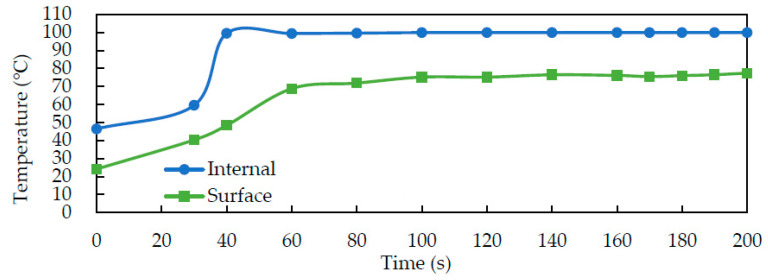
Internal and surface temperature profiles of the solid-state fermented *W. cocos* products during RF drying with an electrode gap of 15 cm. Data are expressed as mean ± SD (*n* = 3).

After pasteurization, the solid-state fermented *W. cocos* products contained about 40% moisture content and required drying steps for storage. A 500 g packet of solid-state fermented *W. cocos* product was laid flat on a plate and dried under an electrode gap of 15 cm under the same conditions as the RF pasteurization treatment. The internal temperature of the sample increased from 45 °C to more than 100 °C after 50 s of RF drying. Due to water evaporation, the internal temperature remained around 100 °C, while the surface temperature increased from 25 °C to more than 60 °C. The surface temperature increased to more than 70 °C after 80 s of RF drying, and it also showed a flat phenomenon, indicating that it entered the latent heat stage of moisture evaporation (Figure 7).

**Figure 8:**
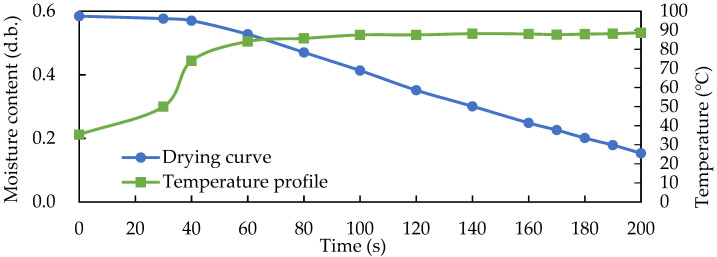
Average temperature profile and drying curve of the solid-state fermented *W. cocos* products during RF drying with an electrode gap of 15 cm. Data are expressed as the mean ± SD (*n* = 3).

Figure 8 shows the average temperature profile and drying curve of the solid-state fermented *W. cocos* product during RF drying. The temperature of the fermented product gradually increased and the moisture content slowly decreased during the first 50 s of RF drying. As a result, the sensible heat condition appeared in this first stage. However, after 50 s of RF drying, the average temperature of the fermented product gradually leveled off at approximately 85 °C and entered the latent heat moisture evaporation condition. The dry base moisture content of the fermented product linearly decreased with the drying time. The linear regression equation was y = − 0.0027x + 0.6806, R^2^ = 0.9982, indicating that the drying rate was constant in this period, with the drying rate at this time being 0.8419 g water/min, and only 200 s of RF drying time reduced the moisture content of the solid-state fermented *W. cocos* product from 40% to 15% or less.

**Figure 9:**
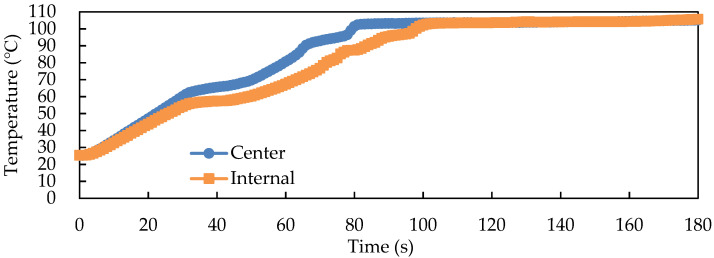
Center and internal temperature profiles of the solid-state fermented *W. cocos* products during continuous RF drying with an electrode gap of 15 cm, pasteurization of 30 s, and drying of 150 s.

If the solid-state fermented *W. cocos* product was used for continuous RF pasteurization and drying treatment, the temperature rising time during the drying process would be reduced by 50 s, thereby reducing the time required for the pasteurization and drying treatment of the fermented product to 180 s, which was extremely fast (Figure 9).

**Figure 10:**
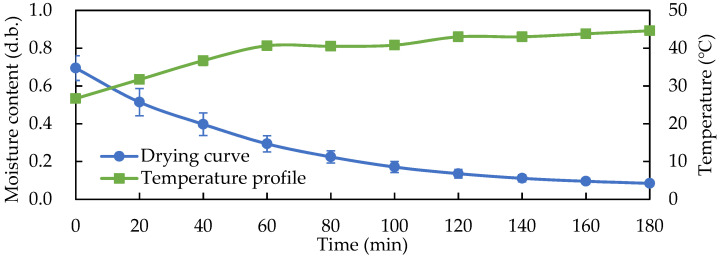
Temperature profile and drying curve during 45 °C hot-air drying of the solid-state fermented *W. cocos* products. Data are expressed as the mean ± SD (*n* = 3).

Compared with a 45 °C hot-air-drying treatment, increasing the surface temperature of the solid-state fermented *W. cocos* products to more than 40 °C took 60 min. The constant drying rate period lasted from 0 to 60 min, the dry base moisture content gradually decreased, and then the drying rate decreased. A total of 100 min was required to reduce the moisture content of the fermented products to less than 15% in 45 °C with the hot-air-drying treatment (Figure 10). Therefore, the rapid overall effect of RF heating was able to quickly dry the solid-state fermented *W. cocos* products, demonstrating a highly efficient drying technology.

Comparing the total drying time required for in-shell walnuts using RF drying, vacuum drying, and hot-air drying, the former was the fastest (138 min), followed by vacuum drying (185 min) and hot-air drying (300 min) [9]. To improve drying efficiency, a combination of RF and hot-air drying may overcome the resistance of both heat transfer and mass transfer.

### 3.3. Active Component and Antioxidant Activity of the Solid-State Fermented W. cocos Mycelia Products

The solid-state fermented *W. cocos* product was subjected to RF pasteurization and drying, 121 °C autoclave and 45 °C hot-air-drying treatment; there was no significant difference in the mycelium, crude polysaccharides, crude triterpenes, total polyphenols, flavonoids, or antioxidant activity of the microwave extract. The solid-state fermented *W. cocos* mycelia product after RF pasteurization and drying contained 5.03% mycelium, 9.83% crude polysaccharides, 4.43% crude triterpenoids, 3.54 mg gallic acid equivalent/g DW of total polyphenols, and 0.38 mg quercetin equivalent/g DW of flavonoids. The supernatant of 50 mg/mL ethanol extract was assayed for antioxidant activity. The results show that the scavenging ability of DPPH free radicals and the chelating ability of ferrous iron were as high as 93.15% and 91.35%, respectively and had similar antioxidant activities to 5 mg/mL of ascorbic acid (93.44% and 91.40%, respectively), and the reducing power was as high as 0.71 (Table 1).

However, in the color analysis, the L* and b* values and whiteness of the solid-state fermented *W. cocos* product by RF pasteurization and drying treatment were significantly higher than those produced by 121 °C autoclaving and the 45 °C hot-air-drying treatment. It showed that the color of the solid-state fermented *W. cocos* product was better preserved through the RF pasteurization and drying process compared with the autoclaving and hot-air-drying process (Table 2).

## 4. Conclusions

In this study, RF heating required only 30 s to stop fermentation and 200 s to dry 500 g of solid-state fermented *W. cocos* product, so it is superior to the traditional sterilization treatment requiring 60 min of autoclaving at 120 °C and followed by the 180 min of 45 °C hot-air-drying method. There was no significant difference in the active components or antioxidant activity between RF and traditional process. RF heating offers a novel method for pasteurizing and drying solid-state fermented fungi products. 

## Figures and Tables

**Table 1:** Effect of different pasteurization and drying methods on the active components and antioxidant activity of the solid-state fermented *W. cocos* products.

Treatment	RF Pasteurization and RF Drying	121 °C Autoclave and 45 °C Hot-Air Drying
Mycelium (%)	5.03 ± 0.12	4.94 ± 0.05
Crude polysaccharides (%)	9.83 ± 0.24	9.35 ± 0.30
Crude triterpenoids (%)	4.43 ± 0.02	4.32 ± 0.01
Total polyphenols (mg gallic acid equivalent/g DW)	3.54 ± 0.21	3.55 ± 0.18
Flavonoids (mg quercetin equivalent/g DW)	0.38 ± 0.03	0.33 ± 0.02
Scavenging DPPH free radicals (%)	93.15 ± 2.46	92.11 ± 0.06
Chelating ferrous ion capacity (%)	91.35 ± 0.33	90.98 ± 0.29
Reducing power	0.71 ± 0.02	0.69 ± 0.03

Data are expressed as the mean ± SD (*n* = 4). A 50 mg/mL sample concentration was used for the antioxidant assay. Means in the same row are not significantly different (*p* > 0.05).

**Table 2:** Effect of different pasteurization and drying methods on the color of the solid-state fermented *W. cocos* product.

Sample	L*	a*	b*	Whiteness (%)
RF pasteurization and RF drying	53.30 ± 0.31 ^a^	9.64 ± 0.03 ^a^	27.70 ± 0.18 ^a^	44.86 ± 0.21 ^a^
121 °C autoclave and hot-air drying	41.74 ± 0.03 ^b^	9.68 ± 0.06 ^a^	22.65 ± 0.15 ^b^	36.75 ± 0.07 ^b^

* Data are expressed as the mean ± SD (*n* = 6). ^a,b^ Means with different superscripts in the same column are significantly different (*p* < 0.05).

## Data Availability

The data presented in this study are available in this article.
